# Factors influencing nurses’ post-traumatic growth during the COVID-19 pandemic: Bayesian network analysis

**DOI:** 10.3389/fpsyt.2023.1163956

**Published:** 2023-08-23

**Authors:** Xi Yao, Junyi Wang, Yingrui Yang, Hongmei Zhang

**Affiliations:** ^1^Henan Eye Institute, Henan Eye Hospital, and Henan Key Laboratory of Ophthalmology and Visual Science, Henan Provincial People’s Hospital, People’s Hospital of Zhengzhou University, People’s Hospital of Henan University, Zhengzhou, Henan, China; ^2^Office of Teaching and Graduate Management, The Fifth Affiliated Hospital of Zhengzhou University, Zhengzhou, Henan, China; ^3^Nursing Department, Henan Provincial People’s Hospital, People’s Hospital of Zhengzhou University, People’s Hospital of Henan University, Zhengzhou, Henan, China

**Keywords:** post-traumatic growth, COVID-19, nurses, influence factors, Bayesian network

## Abstract

**Objective:**

During the COVID-19 pandemic, nurses, especially if females and working in intensive care units or emergencies unit, were much more at risk than other health-workers categories to develop malaise and acute stress symptoms. This study aimed to examine the nurses’ post-traumatic growth and associated influencing factors during the COVID-19 pandemic.

**Methods:**

A cross-sectional study using an online survey was conducted at Henan Provincial People’s Hospital to gather data from nurses. A set of questionnaires was used to measure the participants’ professional identity, organizational support, psychological resilience and post-traumatic growth. Univariate, correlation, and multiple linear regression analyses were used to determine significant factors influencing post-traumatic growth. A theoretical framework based on the Bayesian network was constructed to understand post-traumatic growth and its associated factors comprehensively.

**Results:**

In total, 1,512 nurses participated in the study, and a moderate-to-high level of post-traumatic growth was reported. After screening, the identified variables, including psychological counseling, average daily working hours, average daily sleep duration, professional identity, organizational support, and psychological resilience, were selected to build a Bayesian network model. The results of Bayesian network showed that professional identity and psychological resilience positively affected post-traumatic growth directly, which was particularly pronounced in low- and high-scoring groups. While organizational support positively affected post-traumatic growth indirectly.

**Conclusion:**

Although this study identified a moderate-to-high level of nurses’ post-traumatic growth, proactive measures to improve psychological resilience fostered by professional identity and organizational support should be prioritized by hospitals and nursing managers.

## 1. Introduction

As a public health emergency of international concern, coronavirus disease 2019 (COVID-19) caused by the SARS-CoV-2 virus had a negative effect on people worldwide and posed an unprecedented threat to life and property. The increasing number of daily cases exerted considerable pressure on healthcare systems and caused physiological and psychological suffering among healthcare workers. At the start of the pandemic, with the gradual stringency in governmental measures, a worsening on average of mental health symptoms was reported, but the degree and extent of this worsening and how much may be due to other factors were uncertain. After the 2 months of the pandemic, changes in symptoms varied substantially across studies (1). Considering different populations responded differently to the psychological problems, learning lessons from now was imperative to prepare better strategies for the subsequent waves.

As nurses were required to provide continuous services to patients, which was accompanied by a high risk of infection in addition to high workload demands and irregular working hours, they were more likely to be psychologically overwhelmed (2). During early phases of COVID-19 outbreak, an increased number of working hours, presence of stress symptoms, decline in confidence in the future, and need for psychological support were reported in particular female nurses’ categories among critical care workers (3). According to several systematic reviews and meta-analyses (4–6), stress, anxiety, depression, job burnout, insomnia, and post-traumatic stress syndrome were widespread among healthcare workers. A great deal of sociodemographic, social and occupational factors, such as younger age, decreased social support, longer working time in quarantine areas, working in hospitals with inadequate and insufficient material and human resources, increased workload and lower level of specialized training regarding COVID-19 affected the high levels of nurses’ burnout (7). In addition, during care of patients with COVID-19, the nurses endured a variety of psychological distress, which were related to patients’ death, the disease’s unknown dimensions, the atmosphere of the working environment, professional commitments, and individual characteristics (8). Another study reported a high prevalence of depressive symptoms among nurses, with poor sleep quality, lower optimism of psychological capital, and not visiting friends as independent risk factors (9). Furthermore, under the direct negative influence of occupational stress, the prevalence of insomnia symptoms was comparatively high among nurses, and organizational support and psychological capital as mediating factors partially relieved this chain (10).

As such, both frontline and other nurses suffered significant physiological and psychological damage during the COVID-19 pandemic. Moreover, many nurses were infected and died (11). The nurses’ workforce would be degraded if no external support was provided, which could deliver the health system vulnerable to the crisis. In addition, the long-term influence of the COVID-19 pandemic on mental health was indelible, so significant effort was required to comprehensively understand the full range of its impact. Hu et al. (12) mentioned that frontline nurses’ burnout was negatively correlated with resilience, social support, and work willingness. Hao et al. (13) found that intensive care unit (ICU) nurses with a good professional identity could cope with work pressure better, which improved their work-related wellbeing. Cheng et al. (14) suggested that general self-efficacy and perceived organizational support should be considered to reduce job burnout and increase nurses’ retention. Schierberl Scherr et al. (15) indicated that social support and resilience adequately equipped nurses to adapt to COVID-19 stress. Sarıalioğlu et al. (16) illustrated that the post-traumatic growth levels of nurses with positive COVID-19 polymerase chain reaction test could be improved through providing with psychological and social support.

There are many statistical methods to explore influencing factors, such as Logistic regression analysis, cluster analysis, decision trees, structural equation model, and path analysis, which can not only find relevant influencing factors, but also predict the outcome state. Inevitably, the above methods would expose problems such as complicated results, difficulty in quantification and lack of flexibility when dealing with some complex relationships. However, the combination of Bayesian network graphics and probability just makes up for these deficiencies. On one hand, the relationships between causes, outcomes, and causes and outcomes (including direct and indirect effects) can be analyzed by directed acyclic graph qualitatively. On the other hand, the size of the relationship can be quantified by conditional probability. To comprehensively understanding the post-traumatic growth among nurses and examine the relationships between the associated factors, a Bayesian network model was constructed to take tailored measures to improve the mental states of nurses in hospital organizations and nursing managers.

## 2. Materials and methods

### 2.1. Design, samples, and setting

A cross-sectional study using an online survey was conducted at the Henan Provincial People’s Hospital to gather data from nurses. A set of questionnaires (Supplementary questionnaire) was used to measure the participants’ professional identity (professional identity scale), organizational support (organizational support scale), psychological resilience (psychological resilience scale), and post-traumatic growth (post-traumatic growth scale). A two-dimensional code was sent to the nurses via WeChat. The inclusion criteria were as follows: (1) older than 18 years, (2) registered nurses, (3) involved in clinical work during the COVID-19 pandemic, (4) working in this hospital for at least 1 year, and (5) volunteered to participate in this study. The exclusion criteria were as follows: (1) lactating women, (2) engaged in advanced studies, and (3) working part-time. In total, 1,512 clinical nurses were included in the data analysis.

### 2.2. Instruments

#### 2.2.1. Professional identity scale

The professional identity scale was developed by Liu et al. (17) and includes 30 items rated on a five-point Likert scale (1 = strongly disagree; 5 = strongly agree). The instrument comprises five dimensions: professional identity evaluation (nine items), professional social support (six items), professional social proficiency (six items), dealing with professional frustration (six items), and professional self-reflection (three items). Total scores range from 30 to 150, with higher scores indicating a higher level of professional identity. Cronbach’s was 0.938 for the scale and 0.720–0.911 for the five dimensions.

#### 2.2.2. Organizational support scale

The organizational support scale was developed by Eisenberger and Chen (18, 19), modified by Zuo et al. (20) for nurses. We used the modified scale and added an item, “The hospital does not care about my personal feelings.” The revised scale contains 14 items to measure two aspects of perceived organizational support: emotional (11 items) and instrumental support (three items). Items are rated on a five-point Likert scale (1 = strongly disagree; 5 = strongly agree), and the total scores range from 14 to 70. Higher scores imply stronger organizational support. Cronbach’s alpha was 0.921.

#### 2.2.3. Psychological resilience scale

Nurses’ capacity to bounce back from stressful situations brought about by the COVID-19 pandemic was examined using the Connor-Davidson Resilience Scale. It includes 25 items rated on a five-point Likert scale (0 = never; 4 = always) (21). The scale has five subscales: personal competence and high standards, trust in one’s instincts and tolerance of negative effects, positive acceptance of change and safe relationships, control, and spirituality (22). The total scores range from 0 to 100, with higher scores indicating greater resilience. Cronbach’s alpha was 0.89.

#### 2.2.4. Post-traumatic growth scale

The post-traumatic growth scale was used to measure the positive changes perceived by individuals after a traumatic experience, in other words, the COVID-19 pandemic. It was developed by Tedeschi and Calhoun (23) and translated into Chinese by Wang et al. (24). The scale contains 20 items and 5 dimensions: appreciation of life (six items), new possibilities (four items), relating to others (three items), personal strength (three items), and self-transformation (four items). Answers are rated on a six-point Likert scale (0 = did not experience a change; 5 = experienced a significant change). The total scores range from 0 to 100, with higher scores indicating higher post-traumatic growth. Cronbach’s alpha was 0.874.

### 2.3. Data analysis

IBM SPSS Statistics 23.0 was used to analyze participant characteristics and conduct univariate, correlation, and multiple linear regression analyses. For participant characteristics, frequency and percentage were used for qualitative variables, and mean and standard deviation were used for quantitative variables. To examine differences in post-traumatic growth scores between groups, *t* and *F* tests were adopted for binary and polytomous variables, respectively. Pearson’s correlation analysis was used to explore the relationships between the quantitative variables and post-traumatic growth. Stepwise multiple linear regression including independent variables was used to identify factors influencing post-traumatic growth.

R 4.0.3 bnlearn package was used to build a Bayesian network model (25). A directed acyclic graph containing all nodes shows dependence between variables at the qualitative level, whereas the conditional probability distribution measures relationships at a quantitative level (26). The Max-Min Hill-Climbing algorithm was applied for the former, and the bn.fit() function and Maximum Likelihood Estimation method were applied to obtain conditional probability of nodes for the latter.

Significant level α was set at 0.01 for two sides, expect for 0.05 in the multiple linear regression analysis.

## 3. Results

### 3.1. General description of participants

The study included 1,512 nurses (93.8% women, 6.2% men) with an average age of 32.46 ± 6.04 years (Table 1). Total scores for professional identity, organizational support, psychological resilience, and post-traumatic growth were 120.67 ± 22.24, 54.88 ± 9.27, 73.59 ± 17.30, and 71.75 ± 18.53, respectively.

**TABLE 1:** General description of nurses (*n* = 1,512).

Characteristics	Group	−*x* ± *S*/*n* (%)
Gender	Male	93 (6.2)
	Female	1,419 (93.8)
Age (years)		32.46 ± 6.04
BMI (kg/m^2^)		21.79 ± 2.84
Professional title	Senior	568 (37.6)
	Supervisor	873 (57.7)
	(Co-) Chief superintendent	71 (4.7)
Average monthly earnings (yuan)		10,134.10 ± 3503.14
Marital status	Married	1,054 (69.7)
	Single	458 (30.3)
The number of children	0	558 (36.9)
	1	523 (34.6)
	2 or more	431 (28.5)
Education level	Junior college or below	59 (3.9)
	Undergraduate	1,341 (88.7)
	Postgraduate and above	112 (7.4)
Years of working (years)		10.18 ± 6.87
Average daily working hours (hours)		9.22 ± 1.96
Average daily sleep duration (hours)		6.79 ± 0.93
Day and night shift	Yes	1,209 (80.0)
	No	303 (20.0)
Diabetes	Yes	17 (1.1)
	No	1,495 (98.9)
Hypertension	Yes	35 (2.3)
	No	1,477 (97.7)
Cardiovascular disease	Yes	90 (6.0)
	No	1,422 (94.0)
Psychological changes such as nervousness, anxiety, restlessness and depression	Yes	858 (56.7)
	No	858 (56.7)
The number of COVID-19 front line work engagements		2.16 ± 4.90
Attended training related to COVID-19 patient care	Yes	1,353 (89.5)
	No	159 (10.5)
Received psychological counseling for participating in the COVID-19 work	Yes	294 (19.4)
	No	1,218 (80.6)
Whether the current measures are effective in controlling COVID-19	Yes	1,433 (94.8)
	No	79 (5.2)
Professional identity (points)		120.67 ± 22.24
Organizational support (points)		54.88 ± 9.27
Psychological resilience (points)		73.59 ± 17.30
Post-traumatic growth (points)		71.75 ± 18.53

The score distribution for each scale is shown in Supplementary Figure 1. Approximately 95% of the participants chose neutral or more positive options. However, the spectrum of scores differed for the fourth (“The hospital does not care about my personal feelings”) and ninth (“The hospital does not care about my personal development”) items of the organizational support scale. Scores were evenly distributed among the five choices.

### 3.2. Univariate and correlation analysis

The univariate analysis (Table 2) revealed that professional title (*P* < 0.001), psychological changes (*P* < 0.001), psychological counseling for work during the COVID-19 pandemic (*P* < 0.001), and whether the current measures were effective in controlling COVID-19 (*P* = 0.002) were associated with post-traumatic growth.

**TABLE 2:** Univariate analysis of nurses’ post-traumatic growth.

Characteristics	Group	Score (−*x* ± *S*)	*F*/*t*	*P*
Gender	Male	74.54 ± 18.93	1.496	0.135
	Female	71.57 ± 18.50		
Professional title	Senior	72.77 ± 18.71	9.788	< 0.001
	Supervisor	70.44 ± 18.48		
	(Co-) Chief superintendent	79.76 ± 15.77		
Marital status	Married	71.84 ± 18.40	0.264	0.792
	Single	71.56 ± 18.85		
The number of children	0	71.84 ± 18.57	0.032	0.969
	1	71.82 ± 18.73		
	2 or more	71.56 ± 18.28		
Education level	Junior college or below	73.86 ± 14.71	0.704	0.497
	Undergraduate	71.73 ± 18.63		
	Postgraduate and above	70.92 ± 19.16		
Day and night shift	Yes	71.67 ± 18.84	−0.367	0.714
	No	72.09 ± 17.30		
Diabetes	Yes	70.65 ± 18.41	−0.248	0.804
	No	71.77 ± 18.54		
Hypertension	Yes	66.57 ± 21.07	−1.675	0.094
	No	71.88 ± 18.46		
Cardiovascular disease	Yes	69.78 ± 18.82	−1.043	0.297
	No	71.88 ± 18.51		
Psychological changes such as nervousness, anxiety, restlessness and depression	Yes	68.61 ± 18.49	−7.695	**< 0.001**
	No	75.88 ± 17.78		
Attended training related to COVID-19 patient care	Yes	72.16 ± 18.59	2.483	0.013
	No	68.31 ± 17.72		
Received psychological counseling for participating in the COVID-19 work	Yes	77.49 ± 18.70	5.977	**< 0.001**
	No	70.37 ± 18.23		
Whether the current measures are effective in controlling COVID-19	Yes	72.18 ± 18.24	3.213	**0.002**
	No	64.09 ± 21.96		

The bold values were significant *P* values.

The correlation analysis (Table 3) revealed that average daily working hours (*r* = −0.079, *P* = 0.002), average daily sleep duration (*r* = 0.074, *P* = 0.004), professional identity (*r* = 0.768, *P* < 0.001), organizational support (*r* = 0.726, *P* < 0.001), and psychological resilience (*r* = 0.836, *P* < 0.001) were associated with post-traumatic growth.

**TABLE 3:** Correlation analysis of nurses’ post-traumatic growth.

Characteristic	*r*	*P*
Age (years)	−0.005	0.835
BMI (kg/m^2^)	0.038	0.143
Average monthly earnings (yuan)	−0.019	0.461
Years of working (years)	0.007	0.796
Average daily working hours (hours)	−0.079	**0.002**
Average daily sleep duration (hours)	0.074	**0.004**
The number of COVID-19 front line work engagements	0.044	0.090
Professional identity (points)	0.768	**< 0.001**
Organizational support (points)	0.726	**< 0.001**
Psychological resilience (points)	0.836	**< 0.001**

The bold values were significant *P* values.

### 3.3. Multiple linear regression analysis

After preliminary screening, statistically significant variables were included in the multiple linear regression analysis. The results revealed a significant linear correlation between the independent variables and post-traumatic growth (*F* = 649.019, *P* < 0.001), with 72.1% (*R*^2^ = 0.721) variation in the dependent variable. The regression equation (Table 4) was as follows:

**TABLE 4:** Multiple linear regression analysis of nurses’ post-traumatic growth.

Independent variable	Unstandardized coefficients		Unstandardized coefficients	*t*	*P*	Collinearity statistics	
	*B*	Standard error	β			Tolerance	VIF
Constant	−7.058	3.178	/	−2.221	0.026	/	/
Received psychological counseling	−2.200	0.643	−0.047	−3.420	**0.001**	0.981	1.020
Average daily working hours	0.306	0.131	0.032	2.336	**0.020**	0.968	1.033
Average daily sleep duration	0.601	0.274	0.030	2.192	**0.029**	0.988	1.012
Professional identity	0.152	0.024	0.182	6.357	**< 0.001**	0.226	4.430
Organizational support	0.166	0.052	0.083	3.211	**0.001**	0.277	3.608
Psychological resilience	0.659	0.028	0.615	23.215	**< 0.001**	0.264	3.786

The bold values were significant *P* values.

Post-traumatic growth = −7.058 to 2.200 × received psychological counseling + 0.306 × average daily working hours + 0.601 × average daily sleep duration + 0.152 × professional identity + 0.166 × organizational support + 0.659 × psychological resilience.

Therefore, factors influencing post-traumatic growth were psychological counseling (*t* = −3.420, *P* = 0.001), average daily working hours (*t* = 2.336, *P* = 0.020), average daily sleep duration (*t* = 2.192, *P* = 0.029), professional identity (*t* = 6.357, *P* < 0.001), organizational support (*t* = 3.211, *P* = 0.001), and psychological resilience (*t* = 23.215, *P* < 0.001). These factors were used to build the Bayesian network model.

### 3.4. Bayesian network

**FIGURE 1:**
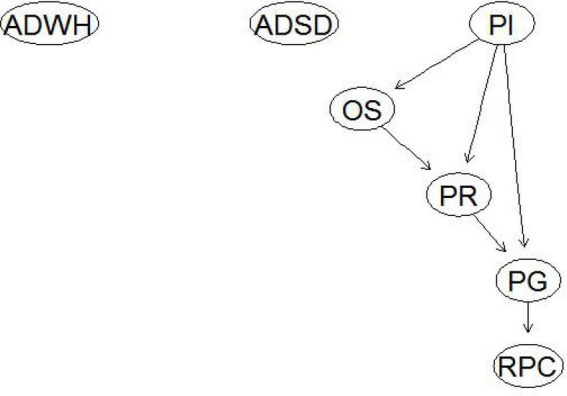
Bayesian network of influencing factors of nurses’ post-traumatic growth. ADWH, average daily working hours; ADSD, average daily sleep duration; RPC, received psychological counseling; PI, professional identity; OS, organizational support; PR, psychological resilience; PG, post-traumatic growth.

Bayesian network node definitions are listed in Supplementary Table 1. Continuous variables were transformed into discrete variables based on data distribution. In the Bayesian network (Figure 1), average daily working hours and average daily sleep duration were independent of the other variables. Professional identity directly affected post-traumatic growth and indirectly affected post-traumatic growth through organizational support and psychological resilience. Moreover, psychological resilience mediated the effect of organizational support on post-traumatic growth. Furthermore, post-traumatic growth influenced psychological counseling.

For the parameter estimation, the accuracies of post-traumatic growth and counseling nodes were 79.4 and 81.3%, respectively. The conditional probabilities for the post-traumatic growth node are presented in Table 5.

**TABLE 5:** The conditional probabilities for node PG.

PI	PR	PG	Conditional probabilities
Low	Low	Low	0.882
Low	Low	Middle	0.117
Low	Low	High	0.001
Low	Middle	Low	0.294
Low	Middle	Middle	0.663
Low	Middle	High	0.043
Low	High	Low	0.177
Low	High	Middle	0.529
Low	High	High	0.294
Middle	Low	Low	0.576
Middle	Low	Middle	0.412
Middle	Low	High	0.012
Middle	Middle	Low	0.125
Middle	Middle	Middle	0.829
Middle	Middle	High	0.046
Middle	High	Low	0.087
Middle	High	Middle	0.425
Middle	High	High	0.488
High	Low	Low	0.429
High	Low	Middle	0.500
High	Low	High	0.071
High	Middle	Low	0.125
High	Middle	Middle	0.659
High	Middle	High	0.216
High	High	Low	0.037
High	High	Middle	0.143
High	High	High	0.820

The probability of a rise in post-traumatic growth increased with professional identity and psychological resilience, which was particularly pronounced in low- and high-scoring groups. The conditional probabilities of the post-traumatic growth node are listed in Table 6. Participants with higher post-traumatic growth scores focused more on their mental health and were more likely to receive counseling.

**TABLE 6:** The conditional probabilities for node RPC.

PG	RPC	Conditional probabilities
Low	Yes	0.131
Low	No	0.869
Middle	Yes	0.181
Middle	No	0.819
High	Yes	0.294
High	No	0.706

## 4. Discussion

The results revealed moderate to high post-traumatic growth. In addition, professional identity, organizational support, and psychological resilience were positively correlated with post-traumatic growth. Moreover, organizational support and psychological resilience mediated the effect of professional identity on post-traumatic growth.

Harmful or distressing events usually result in negative outcomes, such as post-traumatic stress disorder symptoms; however, they can also create positive post-traumatic growth. Nurses who participated in patient care during the COVID-19 pandemic were considered to have experienced trauma. COVID-19 was initially recognized in Wuhan City, China, on 31 December 2019. Since then, nurses had endured multidimensional trauma and mental health problems due to staff shortages, lack of personal protective equipment and medical supplies, and fatigue from long working hours (27). It was vital to help nurses translate these negative experiences into post-traumatic psychological growth, regain confidence in life, and stimulate enthusiasm for work to weaken the long-term adverse effects of the pandemic and return to a normal mental state. To measure the positive changes perceived by individuals after a traumatic experience, in other words, the COVID-19 pandemic, the post-traumatic growth scale was used in our study. The questions of the scale focused more on the participants’ psychological growth after the pandemic, especially the psychological changes brought about by the pandemic. For example, “I think the pandemic has changed my priorities for what’s important in life,” “I think the pandemic has made me more aware of the value of my life,” “I think the pandemic has given me a better understanding of spiritual things” and so on. A moderate-to-high level of post-traumatic growth was reported, which were in line with Cui et al. (28) and Peng et al. (29). Moreover, previous studies reported scores of 43.80 ± 14.65 on the 16-item scale (30), 67.17 ± 14.79 on the 17-item scale (31), and 96.26 ± 21.57 on the 21-item scale (32). In addition to scale, study population, sample size, and year of the study could be potential explanations for the differences. In any case, most participants exhibited post-traumatic growth during the COVID-19 pandemic. These negative experiences helped the participants reflect on their work and find meaning, re-interpret their lives, positively adjust their self-perception, ameliorate the negative effects, and improve life satisfaction (33). Furthermore, care from family and friends, gratitude from patients, relationships with encouraging and considerate coworkers, and support from hospitals played significant roles (34).

The Bayesian network model indicated nurses’ professional identity, organizational support, and psychological resilience were important factors that positively influenced post-traumatic growth. Furthermore, organizational support and psychological resilience mediated the effect of professional identity on post-traumatic growth. Professional identity had three aspects: self (who I am), role (what I do), and context (where I do it), which included the values and beliefs of nurses that guide them thinking, action, and interaction with patients. The items of the professional identity scale focused on the realization of self-worth (both material and spiritual). For example, “Nursing makes me feel worthwhile,” “Nursing allows me and my family to hold valuable medical resources,” “Nursing work allows me to give full play to my abilities and strengths” and so on. These aspects were related to an improved quality of patient care and decreased turnover intention (35). The results indicated that professional identity was relatively high. Participants with higher professional identity were more likely to be keenly interested in the nursing profession, better understand their role and identity, and form positive attitudes toward their work. Work experiences at the frontline enabled nurses to perceived professional benefits, including improved nursing image, social support, and reputation reward (36). All nurses in our study volunteered to work during the COVID-19 pandemic and did not regret their decision. Caring for patients with COVID-19 was meaningful and provided a sense of happiness, accomplishment, and spiritual support (37). In sum, professional identity positively affected organizational support, psychological resilience, and post-traumatic growth.

Organizational support referred to an emotional bond between managers and employees, which was measured by the organizational support scale. The items was mostly the nurses’ perception of the instrumental and emotional support provided by the hospital. For example, “The hospital did its best to provide me with the training I needed for the job,” “The hospital can actively listen to my advice,” “The hospital respect my goals and values” and so on. Obviously, the participants tended to have positive attitudes toward patient care during the COVID-19 pandemic and made efforts to accomplish frontline work if the hospital provided resources, encouragement, and reinforcement. Furthermore, a growing body of research demonstrated significant relationships between organizational support and increased job performance, reduced anxiety (38), intention to stay (39), and perceived professional benefits (14). The participants perceived moderate organizational support, likely due to adequate personal protective equipment, nursing training, and financial reward. The participants were more motivated, satisfied, and resilient and experienced less stress when performing their duties (40). Importantly, as many participants reported feeling that the hospital did not care about their personal feelings and growth, intervention strategies were required to improve perceived care regarding personal feelings and development.

To examine the nurses’ capacity to bounce back from stressful situations brought about by the COVID-19 pandemic, the Connor-Davidson Resilience Scale was used in our study. The questions of the scale focused more on the participants’ psychological attitude when facing the pandemic. For example, “During the pandemic, I think we can adapt to change,” “Whatever happens during the pandemic, I think we can handle it,” “Coping positively with stress makes me feel empowered” and so on. Pre-pandemic, psychological resilience was recognized as a protective factor in safeguarding nurses’ mental health, including protection against stress, depression, and anxiety provoked by disasters or disease outbreaks (41, 42). During the COVID-19 pandemic, psychological resilience was strongly associated with job satisfaction, psychological wellbeing (43), organizational turnover intention, and nurse-assessed quality of care (44). Despite the intense frontline work, our sample exhibited high scores for psychological resilience. Nurses with adequate resilience could flexibly respond to the challenges of COVID-19 pandemic, avoid trauma, and manage their negative emotions to obtain optimal clinical performance and care provision. This study also extended the previous knowledge on the importance of resilience among nurses and supported the argument that positive resilience is linked to increased post-traumatic growth. The present results were consistent with previous research (45), and contributing to a better understanding of nurses’ mental health during the COVID-19 pandemic. The participants achieved growth through the way they responded to the experience. Resilient persons viewed nursing as a unique profession rather than a job, concentrated on development opportunities, and persisted in their careers. They persisted in frontline work, relying on the frequent use of positive skills and methods. As such, to promote post-traumatic growth among nurses, professional identity, organizational support, and psychological resilience must be bolstered and policies tailored to improve the nursing environment at the hospital and social levels were required.

This study had several limitations, and caution should be exercised when interpreting and generalizing the findings. First, the cross-sectional study design did not include longitudinal observations of participants. For example, we could not determine whether participants had psychological issues before the COVID-19 outbreak. Second, continuous variables could not be included in a Bayesian network; therefore, they require discretization. Information stored in the data was missing. Data distribution and prior knowledge were used for continuous variables without specific grouping criteria. If there are excessive nodes or states, the model becomes complex, and the difficulty of calculating the conditional probability increases during parameter learning. Third, all participants were from one hospital, and selection bias cannot be ignored. Finally, as an important factor influencing post-traumatic growth, workplace environment was not investigated because no suitable measurement variable was found. Further research with larger sample sizes, more influencing factors, including various medical institutions and departments, is urgently needed.

## 5. Conclusion

This study provided a new perspective to improve nurses’ post-traumatic growth during the COVID-19 pandemic, which had lasting effects on nurses’ careers. Positive professional identity associated with increased organizational support, promoted psychological resilience, and enhanced post-traumatic growth subsequently. To guide nurses overcome adverse events, the comprehensive post-traumatic recovery plans that help nurses balance the pros and cons of front-line work experience were needed for hospitals, nurse managers, and other healthcare organizations. In view of the constantly changing psychological status caused by a variety of factors, it was recommended to provide close psychological monitoring and long-term psychological counseling for nurses.

## Data availability statement

The raw data supporting the conclusions of this article will be made available by the authors, without undue reservation.

## Author contributions

XY and JW: study conception and design, data analysis and interpretation, and drafting of the article. YY and HZ: data collection. All authors critically revised the article and approved the final version.
