# Supplementary material for: Factors influencing nurses’ post-traumatic growth during the COVID-19 pandemic: Bayesian network analysis
Source: Front Psychiatry. 2023 Aug 23;14:1163956. doi: 10.3389/fpsyt.2023.1163956 (PMC10482097; doi:10.3389/fpsyt.2023.1163956)
Supplement: Supplementary file 1 [file Data_Sheet_1.docx]

**Supplementary Questionnaire** (Chinese and English version)

**关于一线护理人员的创伤后成长研究**

**A study of post-traumatic growth in front-line nurses**

尊敬的女士/先生：

您好！请您仔细阅读每一项叙述，并选择符合您条件和意愿的选项。这是一份学术性问卷，不需要填写个人姓名，您所填的答案仅供整体统计分析之用，绝不个别处理或公开发表，资料完全保密，不对任何人泄露，请您放心填答。十分感谢您的协助以及对本研究的支持！

Dear Madam/Sir:

Hello! Please read each statement carefully and select the option. This is an academic questionnaire, you do not need to fill in the personal name. The answer is only for statistical analysis, will not be individually processed or published. The data will not be disclosed to anyone, please feel free to fill in. Thank you very much for your assistance and support for this study!

**一、一般资料问卷 General information questionnaire**

1. 您的性别：Gender

①男 Male

②女 Female

1. 您的年龄： 岁 Age (years)
2. 您的身高： cm Height (cm)
3. 您的体重： kg Weight (kg)
4. 您的婚姻状况：Marital status

①在婚 Married

②单身 Single

1. 您的孩子个数：The number of children

①0 zero

②1 one

③2个及以上 two or more

1. 您的受教育程度：Education level

①专科及以下 Junior college or below

②本科 Undergraduate

③研究生及以上 Postgraduate and above

1. 您的工作年限： 年 Years of working (years)
2. 您平均每日工作时长： 小时 Average daily working hours (hours)
3. 您平均每日睡眠时长： 小时 Average daily sleep duration (hours)
4. 您是否需要日夜倒班：Day and night shift

①是 Yes

②否 No

1. 您的职称：Professional title

①初级 Senior

②中级 Supervisor

③高级 (Co-) Chief superintendent

1. 您的平均月收入：Average monthly earnings (yuan)

①＜5000

②5000-7999

③8000-9999

④≥10000

1. 您是否患有糖尿病？ Whether you have diabetes?

①是 Yes

②否 No

1. 您是否患有高血压？ Whether you have hypertension?

①是 Yes

②否 No

1. 您是否患有心血管疾病？ Whether you have cardiovascular disease?

①是 Yes

②否 No

1. 疫情以来您是否存在心理问题？如焦虑、抑郁等？

Have you had any psychological changes such as nervousness, anxiety, restlessness and depression?

①是 Yes

②否 No

1. 您有 次新冠肺炎一线工作经历？

How many times have you had front-line work experience with COVID-19?

1. 您是否参加过新冠肺炎病人护理的相关培训？

Have you attended any training related to the care of COVID-19 patients?

①是 Yes

②否 No

1. 您是否因为参加新冠肺炎一线工作接受过心理咨询？

Have you received psychological counseling because of your front-line work?

①是 Yes

②否 No

1. 您认为目前的措施对于控制新冠肺炎是否有效？

Do you think the current measures are effective in controlling COVID-19?

①是 Yes

②否 No

**二、职业认同量表 Professional Identity Scale**

1. 目前护理工作让我感觉有价值/Nursing makes me feel worthwhile now.
2. 目前我可以与周围任何人接近并打交道/I can approach and interact with anyone around me now.
3. 同事的理解与支持使我获得幸福感/My colleagues’ understanding and support during the epidemic made me feel happy.
4. 我深信护理人员有主体选择性和能动性，不仅能适应职业环境，还能创造和优化职业环境/I am convinced that nurses have subjective selectivity and initiative, and can adapt to, create and optimize the professional environment.
5. 我认为护理择业既有必然性也有偶然性，你认可它，才会有所作为/I think the choice of nursing profession has both inevitability and contingency. You accept it, you make a difference.
6. 目前护理工作与我的兴趣、性格较匹配/Nursing work matches my interests and personality.
7. 护士职业使我更容易赢得社会大众的尊重/Being a nurse makes it easier for me to win the respect of the public.
8. 我喜欢与人交流，热衷于寻找与社会接触的机会/I enjoy interacting with people and looking for opportunities for social contact.
9. 医生和管理者的认可能使我获得幸福感/The approval of doctors and managers makes me feel happy.
10. 我感到自己能在目前护理工作中得到锻炼和成长/I feel that I can exercise and grow in my current nursing work.
11. 我认为专心从事本职业的人，一定能从护理职业生涯中获得丰厚回馈/I believe that those who devote themselves to their profession will reap great rewards from a career in nursing.
12. 离开护士职业会给我造成一些情感创伤/Leaving nursing would be emotionally traumatic.
13. 我对每天的护理工作充满了兴趣/I am full of interest in everyday nursing work.
14. 我对自己在临床工作中的社交举止很自信/I’m confident in my social demeanor in clinical work.
15. 护理工作可使我和家人握有宝贵的医疗资源/Nursing allows me and my family to hold valuable medical resources.
16. 临床护理工作中遇到困难时，我会积极正面思考，努力寻找解决方法，不轻言放弃/When I encounter difficulties in clinical nursing work, I will think positively, try to find solutions, and never give up easily.
17. 我经常分析自己工作中的优势和劣势，缩小工作中理想自我与现实自我的差距/I often analyze my strengths and weaknesses in my work to narrow the gap between my ideal self and my real self.
18. 目前从事护士工作令我愉快/I enjoy working as a nurse now.
19. 面对护理工作上的难题时，我通常能找到几个解决方法/When faced with a difficult problem in nursing, I can usually find several solutions.
20. 患者和家属的满意度评价可使我获得幸福感/The satisfaction evaluation of patients and their families makes me feel happy.
21. 我把疫情当垫脚石，将其视为攀登更高目标的磨练/I regard the epidemic as a stepping stone, as a test to climb to a higher goal.
22. 护理工作可使我施展个人的能力和特长/Nursing work allows me to give full play to my abilities and strengths.
23. 我愿意与比自己优秀的人比较，以激励自己/I am willing to compare myself with those who are better than me to motivate myself.
24. 心情不好时，我会通过找好友倾诉、听音乐或参加体育活动等方式调整自己/When I’m in a bad mood, I adjust myself by talking to friends, listening to music, or participating in sports activities.
25. 在一线工作中遇到困难时，如果我尽力去做，总能解决问题/When I encounter difficulties in front-line work, if I try my best, I can always solve the problem.
26. 目前的护理岗位可使我发挥自身能力、获得幸福感/My current nursing position enables me to exert my abilities and gain a sense of happiness.
27. 在工作中即使别人反对我，我仍有办法取得我所要的/I have a way of getting what I want at work even if others oppose me.
28. 遇到困难时，我会与处境比我更困难的人比较/When I encounter difficulties, I compare myself to others who are in more difficult situations than I am.
29. 我有明确的护理职业发展目标/I have clear goals for my nursing career.
30. 我对目前从事护士职业有强烈的责任感/I have a strong sense of responsibility for my current career as a nurse.

1=非常不符合、2=不符合、3=有时符合、4=符合、5=非常符合

1=Strongly disagree, 2= Disagree, 3=Sometimes agree, 4=Agree, 5=Strongly agree

**三、组织支持量表 Organizational Support Scale**

1. 医院重视我在一线工作中的贡献，关心我的幸福度/The hospital valued my contribution to the front-line work and cared about my happiness.
2. 医院关心我的福利/The hospital is concerned about my welfare.
3. 医院能积极听取我的意见/The hospital can actively listen to my advice.
4. 医院不太关心我的个人感受/The hospital didn’t care much about my personal feelings.
5. 当我在临床一线工作中遇到困难时，医院会尽力帮助我/When I encounter difficulties in clinical front-line work, the hospital will try to help me.
6. 当我在生活中遇到困难时，医院会尽力帮助我/When I encounter difficulties in my life, the hospital will try to help me.
7. 医院给我提供晋升的机会/The hospital give me the opportunity to promotion
8. 医院尊重我的目标和价值/The hospital respect my goals and values.
9. 医院不太关心我的个人发展/The hospital didn’t care much about my personal development.
10. 医院关心我的健康（身体及心理）/The hospital cares about my health (both physical and mental).
11. 一线工作期间，医院会尽力为我解决生活和家庭的后顾之忧/During the front-line work, the hospital will try its best to solve the worries of my life and family.
12. 医院尽力为我提供良好的工作环境/The hospital did its best to provide me with a good working environment.
13. 医院尽力为我提供工作所需的工具和信息/The hospital tried to provide me with the tools and information I needed to do my job.
14. 医院尽力为我提供工作所需的相关培训/The hospital did its best to provide me with the training I needed for the job.

1=非常不同意 2=不同意 3=不确定 4=比较同意 5=非常同意

1=Strongly disagree, 2= Disagree, 3=Uncertainly, 4=Agree, 5=Strongly agree

**四、心理弹性问卷 Psychological Resilience Scale**

1. 在疫情期间，我能适应变化/During the pandemic, I can adapt to change.
2. 在目前的工作和生活中，我有亲密、安全的朋友关系/I have close and secure friends in my current work and life.
3. 有时，我感觉到所经历的事情需要命运或上帝的帮忙/Sometimes I feel that what I'm going through needs fate or God’s help.
4. 一线工作时无论发生什么我都能应付/I can handle whatever happens on the front line.
5. 过去工作的成功让我有信心面对挑战/The success of my past work gives me confidence to face challenges.
6. 我能看到事情幽默的一面/I can see the humorous side of things.
7. 积极应对压力使我感到自己是有力量的/Coping positively with stress makes me feel empowered.
8. 经历艰难或疾病后，我往往会很快恢复/After a hard time or illness, I tend to recover quickly.
9. 事情发生总是有原因的/Things happen for a reason.
10. 无论结果怎样，我都会尽自己最大努力/No matter what happens, I'll do my best.
11. 我能实现自己的目标/I can achieve my goals.
12. 即使当事情看起来没什么希望时，我也不会轻言放弃/Even when things seem hopeless, I don't give up.
13. 我知道去哪里寻求帮助/I know where to go for help.
14. 在压力下，我能够集中注意力并清晰思考/Under pressure, I can focus and think clearly.
15. 我喜欢在解决问题时起带头作用/I like to take the lead in dealing with problems.
16. 我不会因失败而气馁/I am not discouraged because of failure.
17. 我认为自己是个坚强有力的人/I consider myself a strong person.
18. 我能做出不寻常的或艰难的决定/I can make unusual or difficult decisions.
19. 我能处理不快乐的事情/I can handle unhappy things.
20. 我不得不按照预感行事/I had to act on a hunch.
21. 我做事情有强烈的目的性/I do things with a strong sense of purpose.
22. 我感觉能掌控自己的生活/I feel in control of my life.
23. 在工作中我喜欢挑战/I like challenges in my work.
24. 我努力工作以达到目标/I work hard to achieve my goal.
25. 我对自己的目前取得工作成绩感到骄傲/I am proud of my achievements so far.

0=从来不 1=很少 2=有时 3=经常 4=一直如此

0=Never, 1=Seldom, 2=Sometimes, 3=Often, 4=Always

**五、创伤后成长评定量表 Post-Traumatic Growth Scale**

经历新冠疫情后，我认为/After the COVID-19 pandemic, I think

1. 它改变了我对生命中重要事物的先后排序/It's changed my priorities for what's important in life.
2. 我对自己的生命价值有了更多的认知/I have a greater appreciation for the value of my life.
3. 我培养了新的兴趣/I developed new interests.
4. 我更多依靠自己的感觉来处理问题/I rely more on my feelings to deal with problems.
5. 我对精神层面的事物有了更好的了解/I have a better understanding of spiritual things.
6. 我明白当我遇到困难时可以依靠他人/I know I can count on others when I am in trouble.
7. 我确立了新的人生道路/I set a new path in life.
8. 我有与他人更亲近的能力/I have the ability to be closer to others.
9. 我更愿意表达我的情绪/I prefer to express my emotions.
10. 我知道我能较好地处理困难/I know I can handle difficulties better.
11. 我能以我的生命做更好的事情/I can do better things with my life.
12. 我更能接受任何事情的最坏结果/I’m better at accepting the worst of everything.
13. 我能更好地珍惜每一天/I can appreciate each day better.
14. 它给我带来了新的机会/It brought me new opportunities.
15. 我对他人有了更多的同情/I have more compassion for others.
16. 我花更多精力在人际关系上/I put more effort into my relationships.
17. 对需要改变的事物，我更倾向于去改变它/For things that need to be changed, I prefer to change it.
18. 我发现自己比想象中的更坚强/I found out I was stronger than I thought.
19. 我体会到人生是多么美好/I realized how beautiful life can be.
20. 我更接受了自己需要他人帮助的感受/I know better what it’s like to need help from others.

0=完全没有 1=非常少 2=少 3=有些 4=多 5=非常多

0=Not at all, 1=Very few, 2=Less, 3= Some, 4=More, 5=Very much
