# Supplementary material for: Factors influencing nurses’ post-traumatic growth during the COVID-19 pandemic: Bayesian network analysis
Source: Front Psychiatry. 2023 Aug 23;14:1163956. doi: 10.3389/fpsyt.2023.1163956 (PMC10482097; doi:10.3389/fpsyt.2023.1163956)
Supplement: Supplementary file 2 [file Data_Sheet_2.docx]

| **a**  **c** | **b**  **d** |
| --- | --- |

**Supplementary FIGURE 1** The score distribution of each scale (a. Professional Identity Scale; b. Organizational Support Scale; c. Psychological Resilience Scale; d. Post-Traumatic Growth Scale)
