# Supplementary material for: Factors influencing nurses’ post-traumatic growth during the COVID-19 pandemic: Bayesian network analysis
Source: Front Psychiatry. 2023 Aug 23;14:1163956. doi: 10.3389/fpsyt.2023.1163956 (PMC10482097; doi:10.3389/fpsyt.2023.1163956)
Supplement: Supplementary file 3 [file Table_1.docx]

**Supplementary TABLE 1** Definition of nodes in Bayesian network

| Nodes | Status |
| --- | --- |
| Received psychological counseling | Received (1=Yes), No received (2=No) |
| Average daily working hours (hours) | 5-7 (1=Short), 8 (2=Normal), 9-18 (3=Long) |
| Average daily sleep duration (hours) | 3-6 (1=Short), 7 (2=Normal), 8-12 (3=Long) |
| Professional identity (points) | 30-113 (1=Low), 114-132 (2=Middle), 133-150 (3=High) |
| Organizational support (points) | 14-55 (1=Low), 56-70 (2=High) |
| Psychological resilience (points) | 0-66 (1=Low), 67-79 (2=Middle), 80-100 (3=High) |
| Post-traumatic growth (points) | 0-61 (1=Low), 62-80 (2=Middle), 81-100 (3=High) |
